# Cost-Effective and Durable Ceramic Membrane: Fabrication and Performance Optimization

**DOI:** 10.3390/membranes15100307

**Published:** 2025-10-09

**Authors:** Ahmed H. El-Shazly, Yomna A. Fahmy

**Affiliations:** 1 Chemical and Petrochemicals Engineering Department, Egypt-Japan University of Science and Technology, Alexandria 21934, Egypt; yomna.ali@ejust.edu.eg; 2 Chemical Engineering Department, Faculty of Engineering, Alexandria University, Alexandria 21561, Egypt

**Keywords:** ceramic membrane, dye removal, wastewater treatment, backwash

## Abstract

The main objective of this work is to develop a cost-effective and durable ceramic membrane for water purification. The low-cost ceramic membrane was fabricated using readily available materials, such as clays, aluminum oxide, and calcium carbonate, The membrane was fabricated by uniaxial pressing at different pressures and sintering temperatures, then tested using a scanning electron microscope (SEM) and XRD. The porosity of the resulting membrane was 38.7%, and the contact angle was 65° indicating good hydrophilicity for filtration applications. The main composition was 70% clay, 25% CaCO_3_, and 5% alumina. The removal % for methylene blue was tested at varying concentrations, achieving up to 99% removal, an initial flux of 496.8 L m^−2^ h^−1^, and an average pore size of 2 µm. Furthermore, the research explores the effect of backwashing cycles and techniques on the membrane long-term performance. The results indicated that washing the membrane for four cycles to cleanness has achieved an improved efficiency of the membrane and % dye rejection. Back washing was achieved using no chemicals; only distilled water and drying were used. A preliminary costs assessment of the production for affordable membrane resulted in a value of 170 USD/m^2^. The findings demonstrate that optimizing backwashing cycles is essential for prolonging the membrane lifespan and lowering operation costs.

## 1. Introduction

For humans, animals, and submerged life, water is a very important component of life on Earth. Around 1% is usable, and the remaining is found as unworkable groundwater sources and glaciers [1]. Currently, there are recent trends to treat wastewater like advanced oxidation [2], electro-coagulation [3], Photocatalysis [4], and membranes [5].

Membrane technologies have grown significantly in the last few years, as they are useful in wastewater treatment. Some of their benefits are that they do not require huge equipment, do not consume energy, and have a low capital cost, as they are an affordable technique, which has also demonstrated an eco-friendly technique for wastewater treatment [6]. Membranes have been reported to be useful in a wide scope besides water treatment, such as gas separation [7], the pharmaceutical industry [8], and the petrochemical industry [9].

Water treatment processes employ several types of membranes. They include microfiltration, ultrafiltration, reverse osmosis, and nanofiltration membranes. The main difference between MF and NF is the pore size, which is greater in MF types; thus, they are used in the removal of microorganisms, while the pore size is smaller in NF, which have an advantage over MF in filtering bacteria and very small macromolecules, such as proteins. RO membranes can remove tiny contaminants, such as salt ions, because of their non-porous characteristics. In the filtration technique, a membrane acts as a selective barrier separating two phases and could regulate the movement of elements or impurities depending on the pore [10].

Typically, there are two types of membranes depending on the material of fabrication, whether it is inorganic like ceramic or metallic or organic like polymeric membranes, and this material determines the physical structure [11].

Polymeric membranes are mainly utilized to separate solids. In addition, they can also separate some pathogens from wastewater. One of their advantages is that they require low energy to use and mainly rely on the pressure difference to remove pollutants from wastewater. Moreover, they create zero sludge that requires additional treatment [12]. The use of pure homopolymers for membrane production resulted in a halt in opportunities for investigating membrane uses in emerging areas, such as energy and nanofiltration in nonaqueous environments [13].

Membranes made from carbon-based nanostructures, including graphene, carbon nanotubes, and other derivatives, represent a category of inorganic membranes that have made a new era in membrane science and technology, showcasing remarkable separation capabilities [14].

These carbon-based materials have notable characteristics, including a high permeability and surface area, suitable pore size, and uniform structure, and therefore have gained greater interest in addressing water pollution [15]. There is great attention to graphene materials, and they are addressed as the new generation of RO films because they have a smooth surface, minimal roughness, and reduced nucleation sites. They are thinner, chemically superior, and ion-selective compared to the active layer in polymeric membranes [16].

Inorganic membranes like ceramic are characterized by excellent thermal and chemical stability, high corrosion resistance, ease of maintenance, and an extended lifespan. Unlike polymeric membranes, they can handle high temperatures and acidic mediums and resist fouling as well as corrosion [17].

They also achieved greater permeate flux due to the hydrophilic surface, the high porosity and the good pore size distribution. Furthermore, they are acknowledged as materials capable of withstanding high oil content and various other contaminants in the feed [18,19].

AOPs and EAOPs have recently garnered significant attention because of their advantageous features (such as no requirement for chemical reagents, straightforward process control, reliable performance, and eco-friendliness), as well as their capacity to degrade dyes, as noted in Merouani et al. regarding the use of sono-photocatalytic degradation for persistent textile dyes [20]. Numerous studies tried to combine membranes with EAOPs (membrane–EAOPs) for wastewater treatment. This process is the usage of catalytic membranes by attaching catalyst particles onto the membrane. This membrane can prevent clustering and acquire the capability to oxidize and break down pollutants while isolating them from water [15].

For example, Ganiyu et al. [21] studied the performance of coupling membrane filtration and the advanced oxidation process (membrane AOPs) for the removal of pharmaceutical residues. This achievement was effective for the removal of dangerous pollutants when compared to the membrane or AOPs alone. Photocatalytic oxidation is a promising technique to also be coupled with a ceramic membrane, as Pt was loaded onto a ceramic membrane with deposited TiO_2_. Under UV irradiation, the deposition of Pt increased the rate of formic acid oxidation by about 2–5 times. The Pt-TiO_2_ catalytic membrane avoided Pt oxidation and allowed it to remain in a metallic state even in the presence of reactive oxygen species. Compared with pure Pt catalytic membranes, Pt-TiO_2_ catalytic membranes have a longer lifespan [15]. Also, combined graphene oxide and highly magnetic Fe_3_O_4_ with TiO_2_ components (TiO_2_-GO-Fe_3_O_4_) loaded onto a flat ceramic membrane was reported to be able to destroy -O-O- to generate HO- by UV irradiation. The degradation of amoxicillin (AMX) revealed a mere 22.3% degradation of AMX in darkness, whereas UV irradiation resulted in an 88.5% degradation of AMX [22].

In general, the most common techniques for the fabrication of ceramic membranes are extrusion [23], pressing [24], slip casting [25], and sol–gel [26].

One of the most common problems facing membranes is fouling, which is caused mainly by the deposition and accumulation of undesired materials on the surface of or inside the membranes. The deposited materials could be dissolved particles, partially soluble organic and inorganic macromolecules and biological microorganisms [27].

Fouling can cause significant harm to the membrane material, which leads to a decline in efficiency. This would shorten the total lifespan of the membrane and increase the necessity for replacements [19].

Fouling may also reduce the efficiency of the process, which raises maintenance expenses and lowers output. Membrane fouling can be controlled using physical cleaning or chemical cleaning according to the fouling type and the type of membrane. Physical cleaning uses only fresh air, water, and clean water. Chemical cleaning can be divided into two categories: CIP (cleaning in place) and CEB (chemical enhanced backwashing), based on their cycle of washing and the concentration of chemicals. CIP is mainly utilized to restrict pollutants that are difficult to remove by CEB cleaning with a reduced amount of chemicals [28].

Numerous studies have reported that ceramic membranes exhibit greater fouling resistance than polymeric membranes. One of them examined the efficiency of four types of ceramic membranes (TiO_2_, ZrO_2_, Al_2_O_3_, and SiC) alongside one polymer membrane (polyether sulfone–polyvinyl pyridine, PES–PVP). They discovered that reversible fouling diminishes in the sequence of polymeric > Al_2_O_3_ > ZrO_2_ > TiO_2_ > SiC, while for irreversible fouling, the order is polymeric > ZrO_2_ > Al_2_O_3_ > TiO_2_ > SiC [29].

Numerous methods, including membrane modification, pretreatment, and cleaning, have been tried to improve membrane performance and limit fouling problems [30,31].

It has been found that reversible fouling treatment (Physical Methods) reduces the membrane permeation rate to 10–30% starting within the short time of operation, and it is crucial to select the type of liquid used to wash according to the membrane type [32]. Backwashing is the most famous physical method where the pressure of operating is slightly higher than the original one; the washing liquid can vary, but using distilled or deionized water is more effective and low cost than other liquids, but it may not be effective for multichannel membranes, as it is hard to ensure the uniform distribution of water flow [33,34].

In this context, the aim of this research is to fabricate a ceramic membrane using low-cost raw materials, such as clay, calcium carbonate, and alumina. Furthermore, the affordable membrane employed to process effluents with methylene blue dye was assessed, alongside an examination of various factors, such as the sintering temperature, pressing pressure, and dye concentration. The reusability of the membrane was studied by applying a unique technique of washing that aims to increase the removal of dye by reducing the pore size by washing with distilled water and heating only.

## 2. Experiment

### 2.1. Membrane Material and Preparation of the Flat Composite

The clay was obtained from AVI-CHEM Laboratories (Mumbai, Maharashtra, India). Chemical composition: Al_2_(OH)_4_Si_2_O_5_; molecular weight: 482.99 g. Its primary components included silicon (Si), oxygen (O), iron (Fe), and aluminum (Al), which is mixed with calcium carbonate (pharmaceutical grade, USP, BP, Ph. Eur., PanReac AppliChem, Darmstadt, Germany) and alumina, alpha grade (Merck KGaA, Darmstadt, Germany).

The three elements were meticulously homogenized in a ball mill for 3 h at 200 rpm to create a consistent mixture (70% clay, 25% CaCO_3_, and 5% Al_2_O_3_). Subsequently, the blend is filtered through a 700 µm mesh.

**Figure 1:**
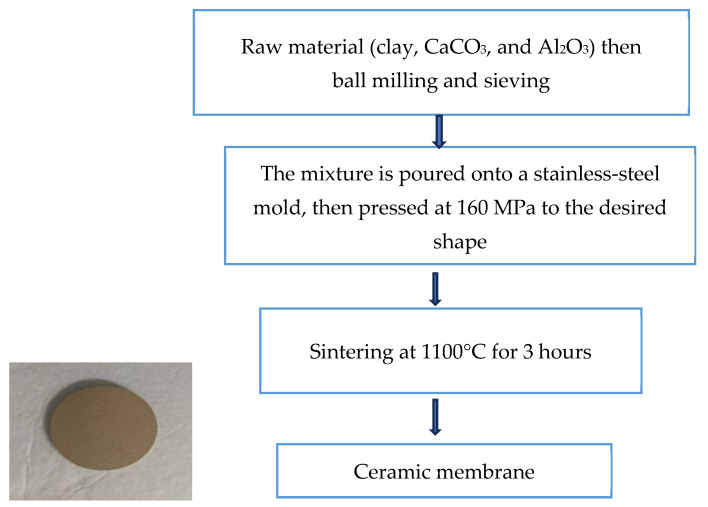
Schematic illustration of the ceramic membrane preparation by the pressing method.

Following this, 2 g of powders were uniaxially pressed into a 25 mm disc using a hydraulic press under a load of 6–8 tons applied over the dye area, corresponding to an effective compaction pressure of approximately 120–160 MPa, held for 60 s. This process yielded a ceramic disc measuring 25 mm in diameter and 1.85 mm in thickness. The discs were sintered in a programmable furnace at 900, 1100, and 1150 °C, and held for 3 h at the target temperature before furnace cooling to room temperature. After this, the ceramic membrane was washed using distilled water at a pressure 0.6 bar for 30 min to remove any excess calcium oxide, then put in the oven at 100 °C for an hour to dry. A schematic of the preparation process is presented in Figure 1, while the general characteristics of the dye used in this study are summarized in Table 1.

### 2.2. Ceramic Membrane Characterization

**Figure 2:**
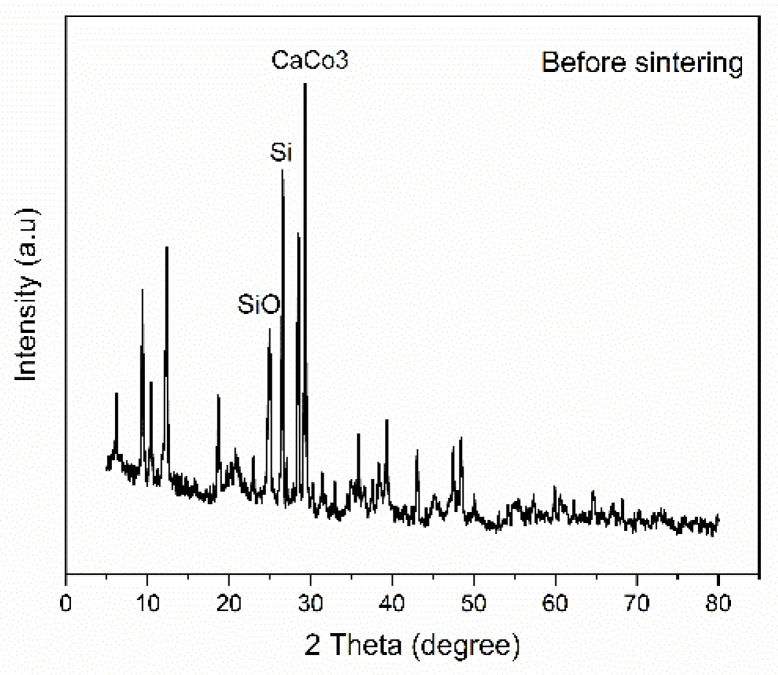
XRD for membrane before sintering.

**Figure 3:**
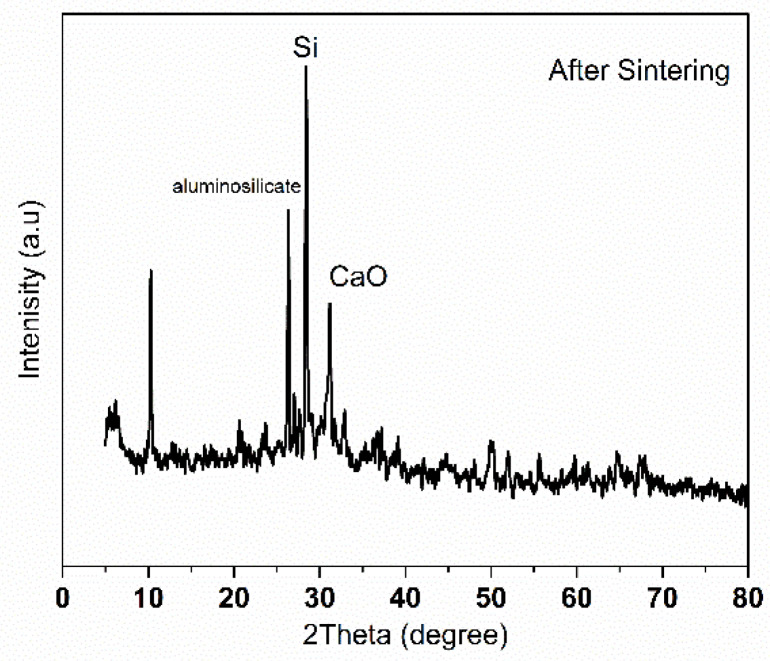
XRD for membrane after sintering.

**X-ray diffraction:** X-ray diffraction analysis was performed using Shimadzu LabX6100 (Kyoto, Japan) for the ceramic membrane before and after sintering at 900 °C for 3 h. Physical treatment of the ceramic membrane is mainly utilized by heat treatment in which there is a change in the structure and composition of clay, especially at high temperatures [35]. The analysis was employed to determine the crystalline and amorphous phases in the raw powder mixture and the fabricated ceramic membrane after sintering. It was also possible to observe in Figure 2 the formation of kaolinite Al_2_Si_2_O_5_(OH)_4_ at 2θ = 10° [36]. The diffraction pattern shows that the main constituents of the clay are silicon and silicon oxide, along with small peaks corresponding to impurities in Figure 2. While being heated, all clay minerals are exposed to a temperature range in which they experience dehydration to different extents. Dehydration causes alterations that can be managed and utilized [36]. After sintering, as in Figure 3, many of the minor peaks disappear, indicating phase transformation. The diffraction peak observed at approximately 2θ = 26° corresponds to the compound Fe_2_Al_4_Si_5_O_18_ [37], which is a complex aluminosilicate phase. This compound is commonly associated with the formation of cordierite-like structures, which can develop during high-temperature sintering of raw materials rich in clay (Si and Al), alumina (Al_2_O_3_), and iron-containing components; the graph also noted that the anorthite (CaO·Al_2_O_3_·2SiO_2_) phase is present in the sintered membranes at 2θ = 22°. The formation of anorthite is attributed to the reaction of CaO with alumina and silica present in the raw material [38], and there is also thermal decomposition of calcium carbonate to calcium oxide, according to the following reaction,

CaCO_3_(s) → CaO(s) + CO(g)

 can be noticed by the vanishing of CaCO_3_ peak and the formation of CaO peak.

**Porosity**: Porosity of membrane was determined using the Archimedes method at different sintering temperatures. Firstly, membrane was weighted dry and then soaked in distilled water for 24 h and the wet weight was measured. The porosity was measured using the formula in (Equation (1)):

(1) $$\mathrm{Porosity}\;=\;\frac{W_{wet}-W_{dry}}{\rho *V}\times 100$$

W_wet_: The weight of soaked membrane after 24 h;
W_dry_: The weight of the original membrane;
ρ: Density of water = 1000 kg/m^3^;
V: Volume of membrane ($\frac{\pi }{4}D^{2}L$).

Table 2 shows that the porosity decreases with the increase in sintering temperature, as explained in Khebli et al. [39], as at elevated temperatures, particles fuse together at their contact points, forming necks. These necks grow with The temperature, reducing the volume of inter-particle voids, and higher temperatures promote grain boundary movement, causing smaller pores to shrink or close entirely, also clarified by the compaction of the body facilitating partial elimination of porosity at elevated temperatures [40].

**Figure 4:**
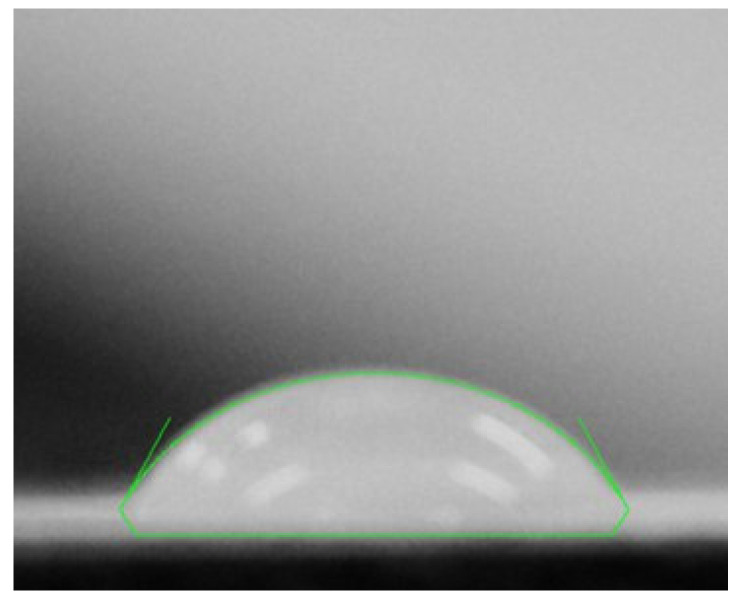
Contact angle image.

**Contact angle**: Contact angle analysis was used to measure the wettability of the membrane surface. A droplet of water was placed onto the membrane and captured using a camera. The image in Figure 4 was analyzed using software Image J (version 1.54g; National Institutes of Health, Bethesda, MD, USA), yielding a value of 65°. This result indicates that the membrane is hydrophilic, which is favourable for dye removal applications.

**Figure 5:**
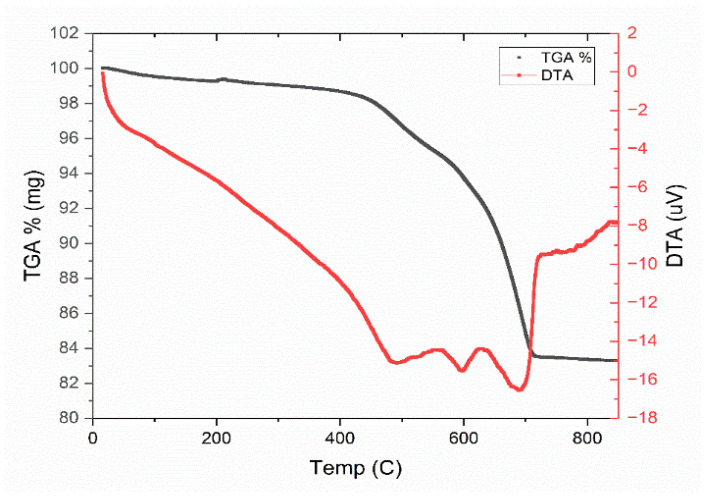
TGA and DTA of powder.

**Thermogravimetric analysis**: Thermal analysis was conducted mainly to identify the major weight loss that occurs during sintering, as temperature is a key parameter, that significantly influences the membrane characteristics. The temperature range of TGA and DTA for the raw material of the membrane was from 15 °C to 900 °C under a nitrogen atmosphere and a 10 °C/min temperature rate, as in Figure 5. The total weight loss of the powder mixture is calculated to be 16.8%. The first low weight loss of 0.72 wt.% corresponding to the first 200 °C is due to the elimination of free and absorbed water [41]. The second effect is seen in the TGA results as a 2.1 wt.% loss at 460 °C, which corresponds to the dihydroxylation of clay [42], and this was also observed in the endothermic peak in the DTA plot. The major weight loss of 16 wt.% observed between 400 and 700 °C resulted from the decomposition of calcium carbonate to calcium oxide and carbon dioxide [43], and this can be confirmed by the strong endothermic peak appeared at 700 °C in the DTA curve. Beyond 700 °C the mass stabilizes, indicating no further decomposition, and the ceramic structure is thermally stable at this point.

**Figure 6:**
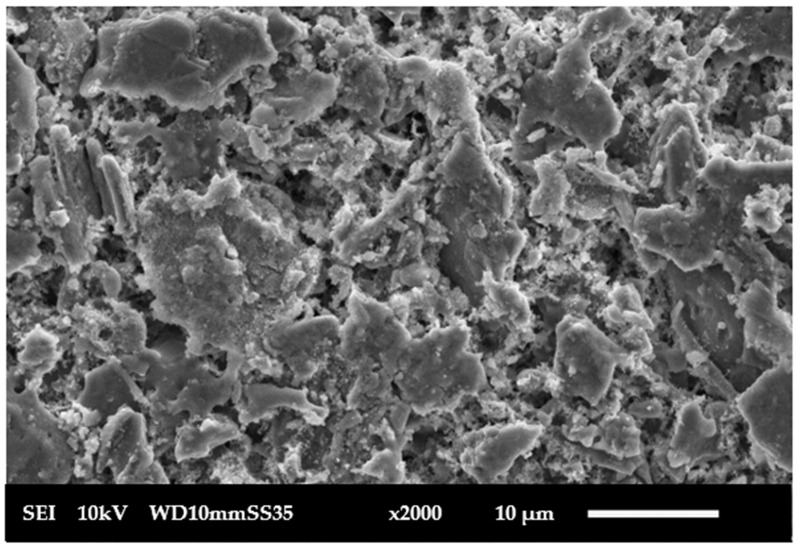
Dry ceramic membrane.

**Figure 7:**
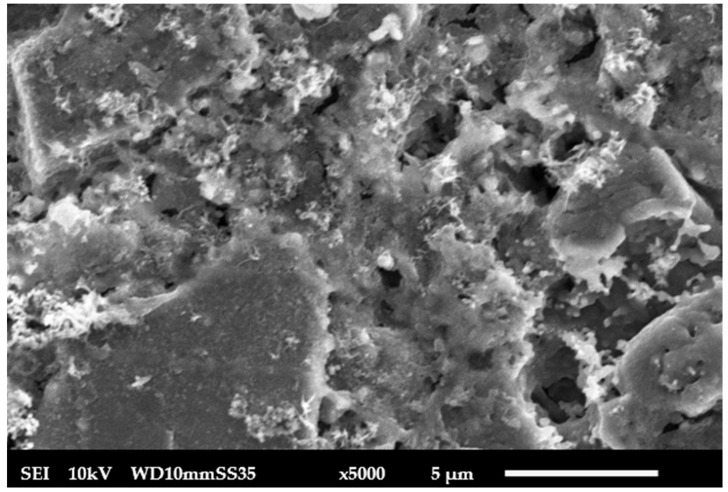
Ceramic membrane after passing dye for 100 min.

**Figure 8:**
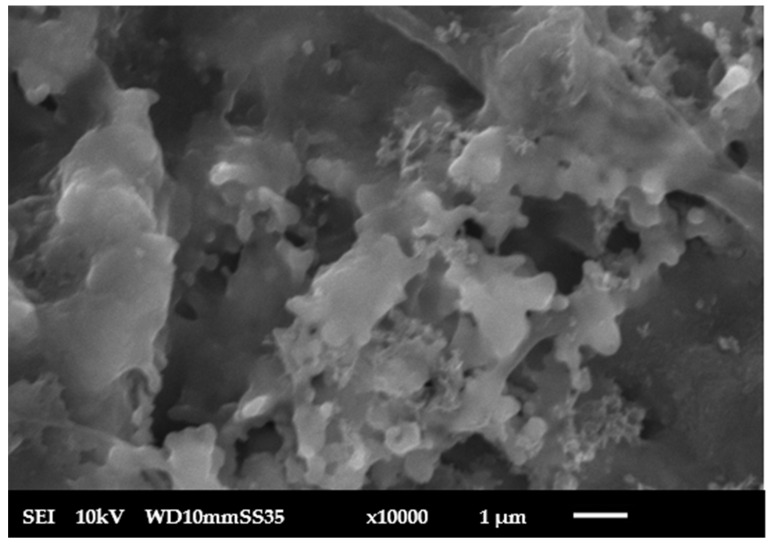
Ceramic membrane after 5 times washing.

**Scanning electron microscopy**: SEM was used by JEOL JSM 6010-LV, Tokyo, Japan, to examine the top surface of the fabricated membrane, as in Figure 6, pressed at 160 MPa and sintered at 1100 °C. The SEM micrographs revealed that the membrane surfaces were homogeneous and free of cracks. SEM micrographs were processed using ImageJ (version 1.54g; National Institutes of Health, Bethesda, MD, USA). Images were first converted to 8-bit grayscale and subjected to background subtraction. Thresholding was applied using the Otsu automatic method to clearly distinguish pores from the solid matrix. The “Analyse Particles” function was then used to measure the equivalent circular diameter (ECD) of each pore. For each membrane, at least 50 pores from three representative micrographs at different magnifications were analyzed. After dye filtration, the membrane surface was examined again using SEM, as in Figure 7. The image shows a decline in visible porosity due to pore blockage by methylene blue dye. The initial average pore size was 2 µm, which was reduced to an average 0.8 µm after 4 cycles of operation and washing for the fifth time, as in Figure 8. The SEM images also revealed partial grain necking with interconnected pores, characteristic of partially sintered ceramic supports where full densification is intentionally avoided to preserve permeability. The pore structure remained mechanically stable after repeated operation, as no evidence of cracks or particle detachment was observed. The reduction in pore size after 5 times of washing indicates also that the structure is mechanically stable, and the microstructure provides a suitable balance between porosity and stability for subsequent functionalization and filtration performance.

### 2.3. Experimental Procedure

**Figure 9:**
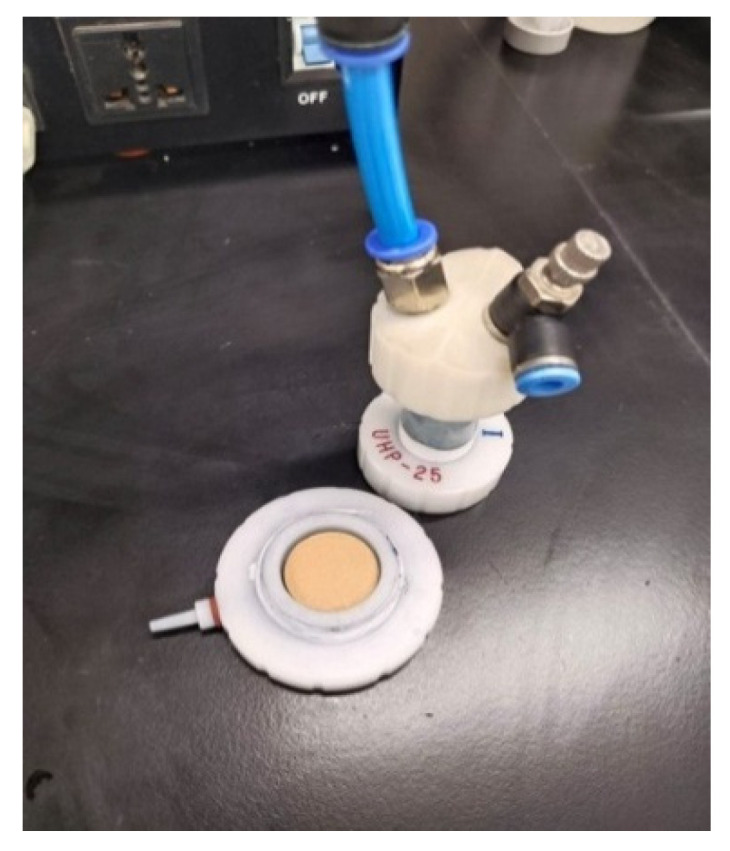
Amicon cell.

**Figure 10:**
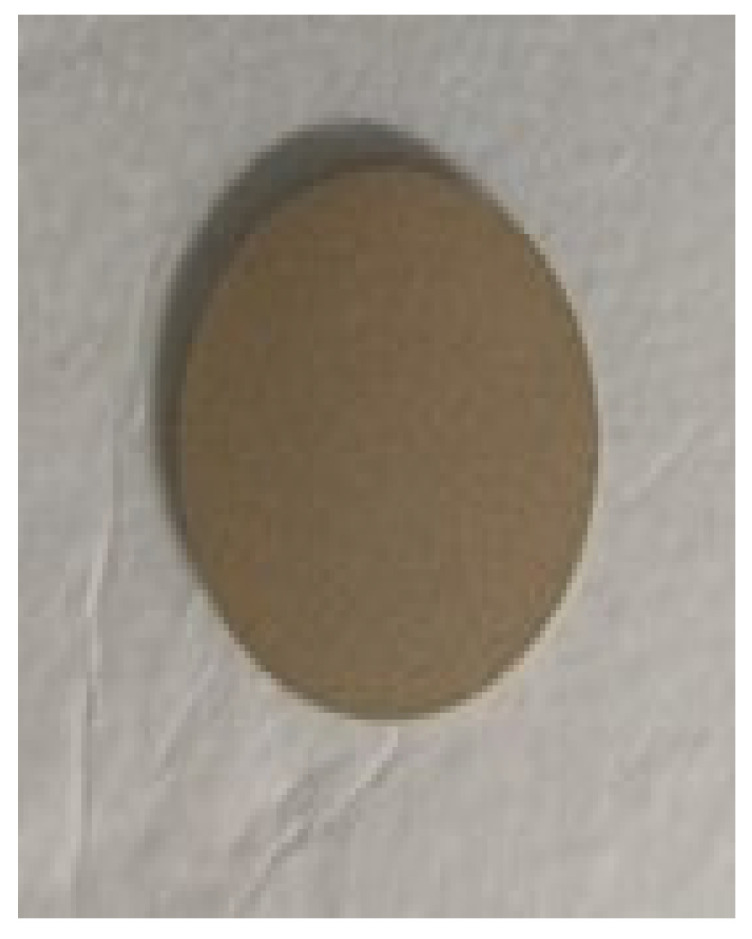
Fresh membrane.

**Figure 11:**
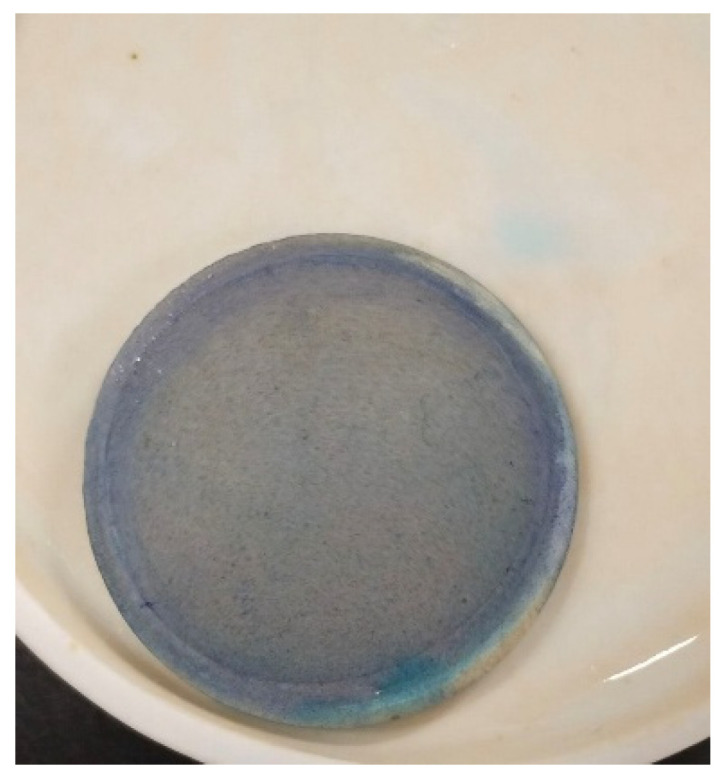
Mmbrane after passing dye.

**Figure 12:**
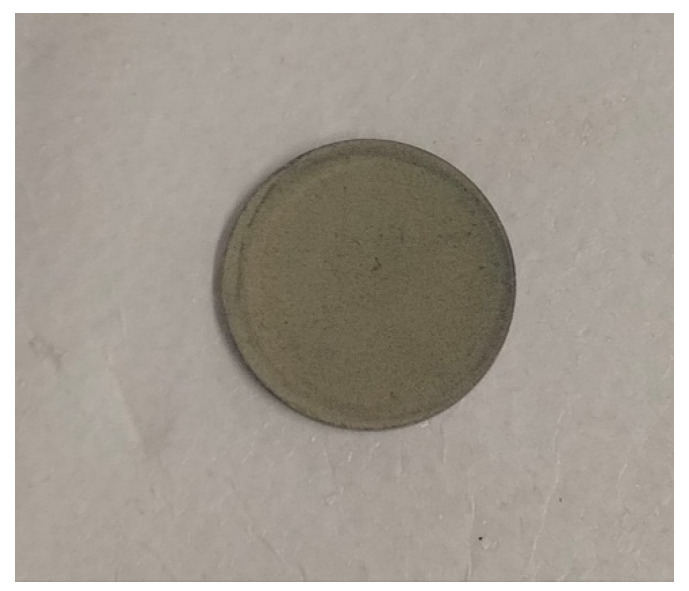
Membrane after washing.

**Figure 13:**
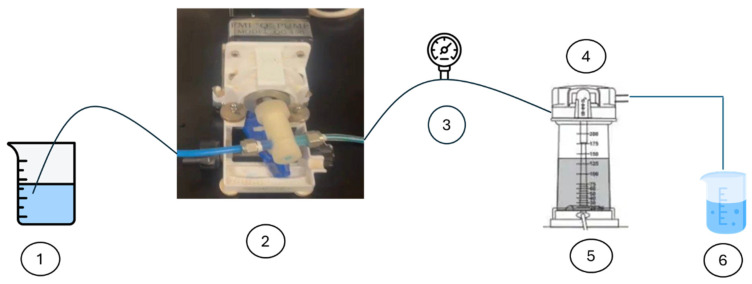
Set up diagram of the system. 1: Feed; 2: pump; 3: pressure metre; 4: Amicon cell; 5: permeate; and 6: Recycled tank.

To prepare a feed solution, 1 L of distilled water was mixed with dye in QVF glass vessels to prepare different concentrations. The water feed was coming from the vessel feed using a piston pump, FMI pump model QG 150, to enter the membrane (ceramic filter) in the Amicon cell, Figure 9,at a fixed pressure of 0.6 bar by using a bypass stream to control the pressure by removing some dye from the feed before passing it into the membrane and collecting it in a recycled tank. The permeate was examined by means of a UV spectrophotometer at a wavelength of 664 nm, dyes concentrations in the feed and permeate solutions were examined within 100 min, then the membrane was dried in an oven at 100 °C for 1 h to remove excess water, then weighed. Fresh distilled water at room temperature was for regeneration of the membrane; the permeate was examined using a UV spectrophotometer until the peak of methylene blue dye disappeared, which took from 5 to 6 h for the first wash. The fresh membrane after passing dye then washing is shown in Figure 10, Figure 11 and Figure 12, respectively. The set up of the filtration system is illustrated in Figure 13.

The process of filtration in this work is mainly by size exclusion through pores and partial adsorption on the surface of functional groups of alumina and clay; there is no catalytic degradation reaction here, as the raw materials are chemically inert at the applied condition. This explains the constant weight of the membrane before and after the process as well as the decline in removal of the dye over time, which can be restored by washing and drying; this confirms the removal here is physical rather than catalytic.

Removal percentage was calculated using Equation (2):

(2) $$R(\%)=\frac{\left(Co-C\right)}{Co}\times 100$$

where R%: removal efficiency (%); C_o_: initial concentration (mg/L); and C: final concentration (mg/L).

The permeate flow of each membrane was calculated using Equation (3), based on the collected volume, collection time, and the effective area of the ceramic membrane:

(3) $$J=\frac{V}{A\cdot \Delta t}$$

where J: water flux (L/m^2^·h); V: permeate volume (L); A: membrane area (m^2^); and Δt: permeation time (hours).

## 3. Results

### 3.1. Influence of Feed Concentration

**Figure 14:**
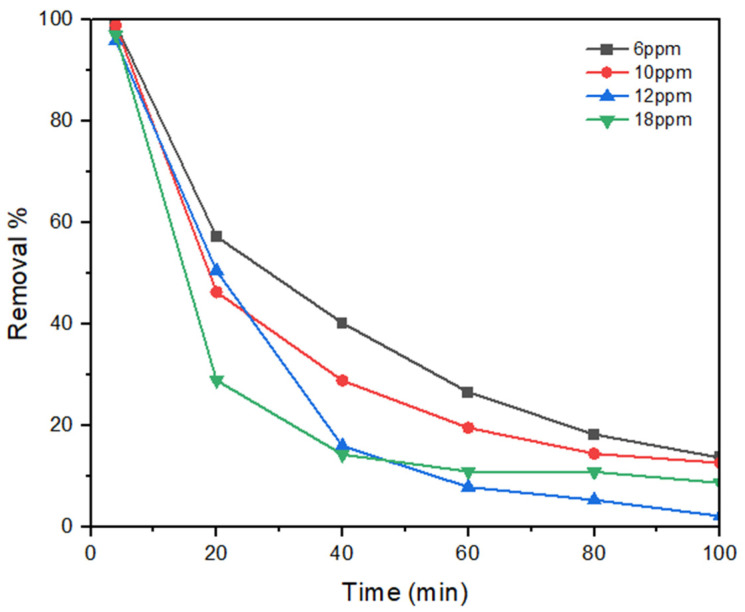
Influence of feed concentration.

The influence of the feed dye concentration was examined under room temperature, an average transmembrane pressure of 0.6 bar, a sintering temperature of 1100 °C, and a feed flow rate of 4 mL/min (Figure 14). As the feed dye concentration increased, a decrease in the removal efficiency was observed. At lower concentrations, the membrane surface provides abundant active sites, leading to a high initial rejection. However, at higher concentrations, these active sites become saturated more quickly, and fouling develops faster, which reduces the rejection percentage over time. Specifically, when the dye concentration increased from 6 to 18 mg/L, the initial rejection dropped slightly from 99% to 97%. After 100 min of operation, the decline was more pronounced, with rejection decreasing from 14% at 6 mg/L to 8% at 18 mg/L. This can be explained by the fact that at elevated pollutant concentrations, there is an increase in concentration polarization that leads to a decline in flux and the rejection of pollutants [44].

### 3.2. Influence of Pressing Pressure

**Figure 15:**
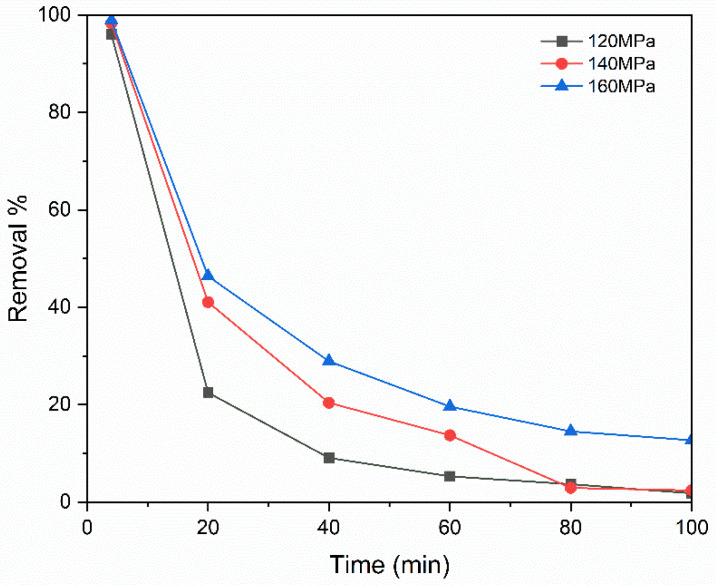
Effect of pressure of pressing.

Figure 15 illustrates the effectiveness of methylene blue rejection at various operation pressures. Every experiment was conducted at room temperature with a dye feed flow rate of 4 mL/min, a sintering temperature of 1100 °C, an average flow pressure of 0.6 bar, and a dye concentration of 10 ppm. Over the course of the trial, it was found that the removal efficiency rose as the pressure increased. This enhancement in performance may be attributed to the reduction in interparticle voids as the moulding pressure increases, which leads to a decrease in the average pore size [45]. Specifically, the removal efficiency improved from 96% to 99% when the moulding pressure increased from 120 MPa to 160 MPa. Also, compaction pressure significantly influences grain growth and microstructural uniformity. Research indicated that optimal pressures promote homogeneous grain structures, which are crucial for the material’s functional properties [46].

### 3.3. Influence of Temperature of Sintering

**Figure 16:**
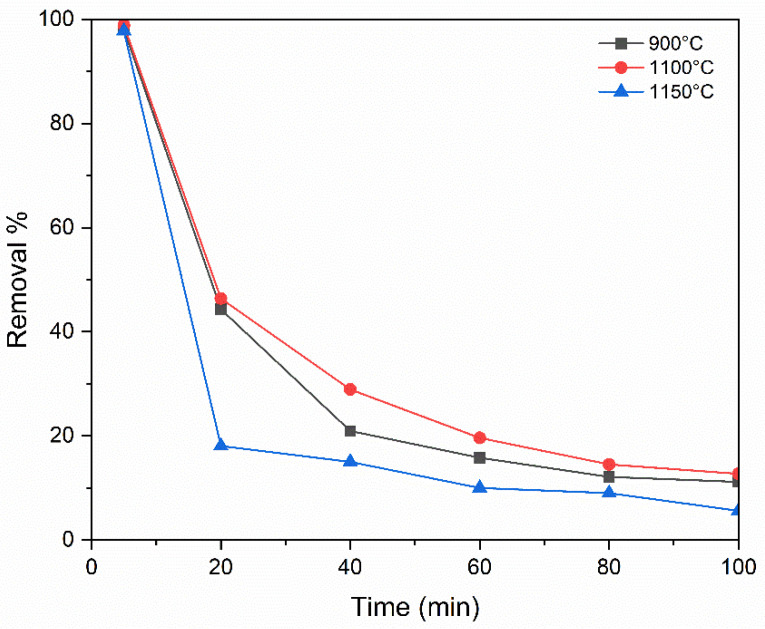
Influence of temperature of sintering.

Sintering of the membrane was performed at different temperatures to study the rejection of the dye at a concentration of 10 ppm and a feed flow rate of 4 mL/min with 0.6 bar operating pressure. The rejection increased by increasing the temperature but to an appropriate level, which confirms a significant structural modification within the porous ceramic matrix [47]. As in Figure 16, at the beginning of the experiment, the removal efficiencies were nearly identical for all membranes. However, after 20 min, a significant decline was observed in the membrane sintered at 1150 °C, where the efficiency dropped to lower than 20%, compared to 46% for the membrane sintered at 1100 °C. This notable difference happened due to the reduction in porosity that occurs at higher sintering temperatures, as is illustrated in Table 2. At 900 °C the rejection after was less than 44%, which is lower than the one sintered at 1100 °C, and this is likely due to the decline in pore size by raising the temperature [48]. These results indicate that higher sintering temperatures enhance the densification of the ceramic structure, which can lead to strengthening the bonding between particles and improving the mechanical integrity, followed by a reduction in pore size that may enhance dye rejection by providing a tighter filtration barrier, thus contributing to the observed improvement in removal efficiency. The time of sintering is also an important parameter in the formation of pores, as when the sintering time is prolonged, grain growth and neck formation become more pronounced, leading to the consolidation and progressive densification of the ceramic matrix. However, complete densification does not always take place during sintering [49]. The incomplete elimination of pores between grains contributes to the presence of residual porosity in the final structure. In addition to temperature, the sintering time is defined as the duration at peak temperature during the sintering process, which influences the extent of material densification but can lead to microstructural coarsening if prolonged. Typically, industrial sintering times are as short as 10 min at the peak temperature, as longer times can be counterproductive [50]. In this study, a fixed duration of 3 h was chosen at each sintering temperature (900, 1100, and 1150 °C) to ensure consistent microstructural evolution for comparison.

### 3.4. Backwash and Regeneration of the Membrane

**Figure 17:**
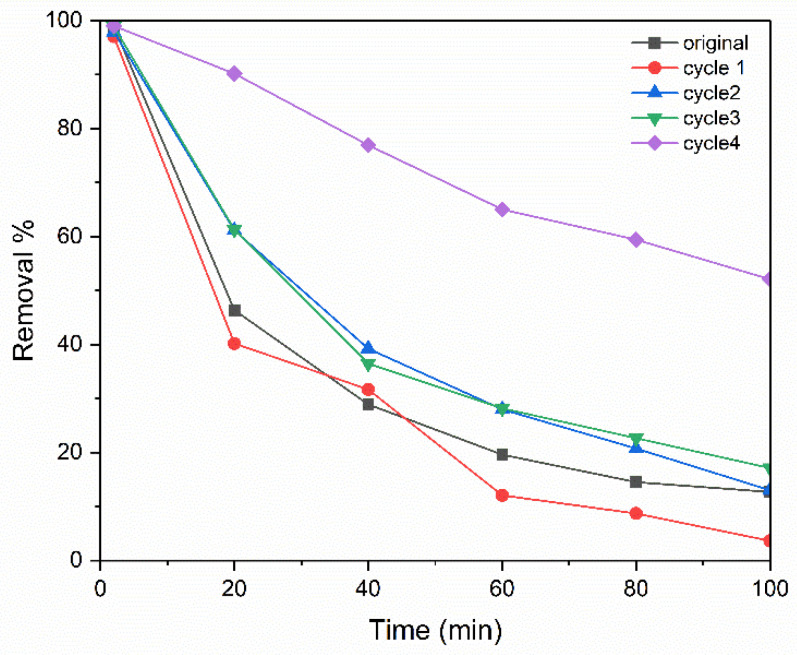
Effect of washing on efficiency.

**Figure 18:**
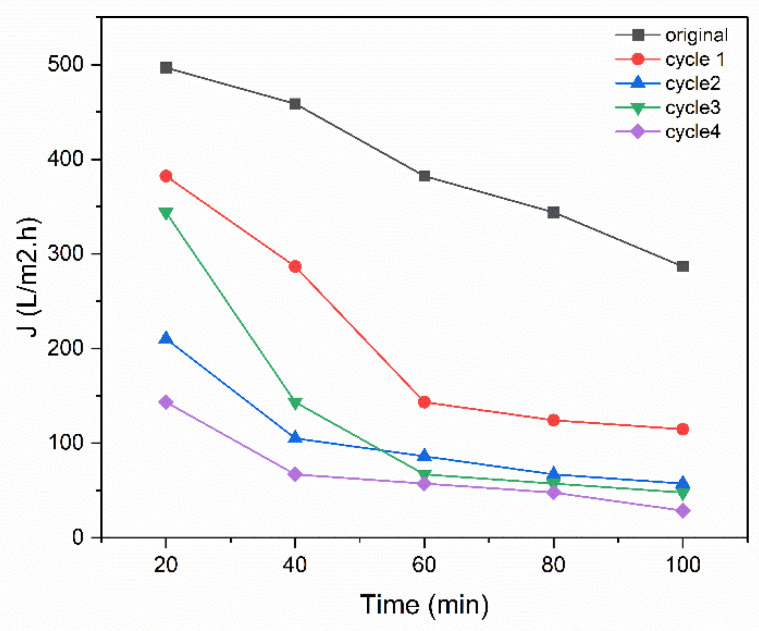
Effect of washing cycles on flux.

Regeneration of the membrane was completed after passing the dye for 100 min by washing the membrane with fresh distilled water at the back side of the membrane with an operating pressure higher than the original one, which was 0.8 bar, and the same direction of flow. Following this, the membrane was dried in an oven at 100 °C for one hour to remove moisture and, stabilize its structure, then membrane was washed to remove any accumulated dye until the permeate had no methylene blue, and the permeate was analyzed using the UV. Afterward, the membrane was dried in an oven at 100 °C for 1 h to remove residual moisture. The regenerated membrane was subsequently tested by repeating the dye filtration for another 100 min. This cycle of dye filtration followed by washing and drying was repeated four times. After each cycle, measurements were taken for dye removal efficiency, washing time, flux, and pore size. The time for washing in the first cycle was 6 h, which gradually increased with each cycle until it reached 18 h after four cycles of operation. The results in Figure 17 show a progressive increase in removal efficiency over the cycles. Initially, during the first 20 min of operation, the removal efficiency was 46% for the original membrane. After the first regeneration cycle, it slightly decreased to 40%, likely due to residual contamination, which would be removed by increasing the number of cycles. However, in subsequent cycles, the removal efficiency increased significantly to 61% and then 90% in the third cycle. This improvement can be attributed to the progressive pore size reduction from 2 to 0.8 µm, resulting from microstructural and surface chemistry changes, such as clay shrinkage when a clay body that has been hydrated is dried [51,52]. Drying at 100 °C likely helps in stabilizing or realigning the microstructure of the clay matrix, causing pore shrinkage or a redistribution that favours size exclusion or stronger capillary forces, such as when pores become wet and are then dried. As evaporation occurs, grains move closer together, yielding body shrinkage to balance the volume of water, which has been removed [53]; as well, washing can remove residual dye and contaminants that may block active sites [54]. This is also partly due to the enhanced hydrophilicity of the alumina/clay matrix, as α-alumina surfaces exhibit superhydrophilicity when hydroxyl groups (aluminols) are exposed, as they tightly bind interfacial water. Washing likely removes residual contaminants and re-exposes these hydroxyl groups, thereby increasing hydrophilicity and promoting the formation of hydrated pore walls, which reduces the effective pore diameter [55]. This effect is followed by a decrease in flux, as it declined from 496.81 to 143.3 L/m^2^·h from the original trial to the fourth cycle in the first 20 min, as shown in Figure 18 of the operation, and this decrease confirms the reduction in the pore size.

This regeneration technique highlights the effect of washing combined with drying. As shown in Table 3, the newly developed membrane, when regenerated using the proposed technique, showed a sustained performance across multiple cycles, reinforcing its potential for reuse and sustainability in dye filtration applications.

### 3.5. Cost Analysis

The cost of manufacturing ceramic membranes in the industrial sector high. For example, it can vary from 500 USD/m^2^ to 1000 USD/m^2^ for a porous tubular ceramic membrane made of α-alumina, with average pore sizes between 1000 and 6000 nm [58], which are relatively high, so there are too many trials to reduce this cost, especially for water purification applications. The estimated cost of the kaolin-based ceramic membrane with pore sizes from 550 and 810 nm was 220 USD/m^2^ [59]. In our study, the fabrication cost of the developed ceramic membrane (including raw materials and processing energy) is approximately 170 USD/m^2^. This is comparatively lower than values reported for many commercial and laboratory-scale ceramic membranes (Table 4). Moreover, our membranes can be reused multiple times with increasing efficiency, further enhancing their cost-effectiveness.

## 4. Conclusions

In this study, a low-cost ceramic membrane was created by dry uniaxial compaction and tested on a laboratory scale for treating methylene blue dye. The membrane was sintered at 1100 °C and had a porosity of 38.7%, an average pore size of 2 µm, a contact angle of 65°, and an initial flux of 496.81 L/m^2^ h. An up to 99% rejection of dye was achieved, and this membrane showed unexpected progressive efficiency by the new technique of washing, including distilled water and drying only, which helped to tighten the pore to reach 0.8 µm after four4 cycles of operation and cleaning, microstructural realignment, enhanced surface adsorption, and fouling-layer effects, despite a concurrent flux decline to 143.3 L/m^2^·h. This enhancement is attributed to microstructural realignment, which favours size exclusion and adsorption mechanisms. The fabrication cost of the membrane was estimated to be approximately 170 USD/m^2^, which is considerably lower than that of conventional ceramic membranes. Combined with its regeneration ability and chemical-free cleaning, this approach significantly extends the membrane’s lifetime while reducing operating costs and the environmental impact, making it a promising candidate for small-scale industrial wastewater treatment units. These findings suggest that optimizing the pore structure and backwashing strategies can further improve the long-term performance, providing a durable, cost-effective, and sustainable solution for water purification applications.

## Figures and Tables

**Table 1:** General characteristics of methylene blue dye.

Parameter	Value
Chemical formula	C_16_H_18_CiN_3_S_4_·H_2_O
Molecular weight (g/mol)	319.85
Absorption maxima (nm)	664

**Table 2:** Porosity at varying sintering temperature.

Sintering Temperature °C	Porosity%
900	39.8
1100	38.7
1150	37.1

**Table 3:** Comparison of backwash cycles on efficiency.

Material of Fabrication	Effect of Backwash on Efficiency	Reference
Ceramic membrane of clay, calcium carbonate, and alumina	Increased by washing	This work
Ceramic of clay, chamotte, calcium carbonate, and potato starch	Remained unchanged after the first backwash and then declined from 90% to 82% after four cycles	[56]
Hydrophilic PES membranes	Gradual decline in efficiency with each filtration cycle	[57]

**Table 4:** Cost of different clay based on low-cost ceramic membranes.

Raw Material of Fabrication	Total Cost of Production	Reference
Clay, calcium carbonate, and alumina	170 USD/m^2^	This work
Flay ash, quartz, and calcium carbonate	250 USD/m^2^	[60]
Chocobofe clay, kaolin, magnesite concentrate, and starch	233.55 USD/m^2^	[36]

## Data Availability

The authors confirm that the data supporting the findings of this study are available within the article.
